# The detectability paradox: bilingual medical report generation with open-weight models and the limits of human oversight

**DOI:** 10.1093/jamia/ocag070

**Published:** 2026-05-08

**Authors:** Hossein Rouhizadeh, Abiram Sandralegar, Anthony Yazdani, Weibo Feng, Oren Schreier, Yonnou Ahn-Kim, Assiya Sirbal, Valentino Pirelli, Rui Yang, Lukas Sveikata, Elena Tessitore, Nan Liu, Philippe Bijlenga, Douglas Teodoro

**Affiliations:** Department of Radiology and Medical Informatics, Faculty of Medicine, University of Geneva, Geneva, 1202, Switzerland; Division of Neurosurgery, Geneva University Hospitals, Geneva, 1211, Switzerland; Department of Radiology and Medical Informatics, Faculty of Medicine, University of Geneva, Geneva, 1202, Switzerland; Department of Radiology and Medical Informatics, Faculty of Medicine, University of Geneva, Geneva, 1202, Switzerland; Department of Radiology and Medical Informatics, Faculty of Medicine, University of Geneva, Geneva, 1202, Switzerland; Faculty of Medicine, University of Geneva, Geneva, 1211, Switzerland; Faculty of Medicine, University of Geneva, Geneva, 1211, Switzerland; Faculty of Medicine, University of Geneva, Geneva, 1211, Switzerland; Centre for Quantitative Medicine, Duke-NUS Medical School, Singapore, 169857, Singapore; Division of Neurology, Department of Clinical Neurosciences, Geneva University Hospitals and Faculty of Medicine, Geneva, 1211, Switzerland; Cardiology Division, Internal Medicine Department, Geneva University Hospitals, Geneva, 1211, Switzerland; Centre for Quantitative Medicine, Duke-NUS Medical School, Singapore, 169857, Singapore; Division of Neurosurgery, Geneva University Hospitals, Geneva, 1211, Switzerland; Department of Radiology and Medical Informatics, Faculty of Medicine, University of Geneva, Geneva, 1202, Switzerland

**Keywords:** large language models (LLMs), medical documentation, automated report generation, multilingual medical reports, electronic health records (EHRs)

## Abstract

**Objectives:**

The automation of medical report generation using large language models (LLMs) could significantly reduce physicians’ documentation burden while enhancing healthcare efficiency. However, the misuse of generative artificial intelligence in medical reporting can lead to important safety risks for patients. We addressed 2 questions: (1) What is the quality of medical reports generated by LLMs in English and French? and (2) Can we distinguish between human-written and LLM-generated medical reports?

**Materials and methods:**

We evaluated the quality of reports generated by several multilingual, open-weight LLMs using text similarity metrics on 4212 medical reports in English and French across multiple specialties. A bilingual expert panel of certified physicians (*n* = 4) and medical residents (*n *= 5) scored accuracy, fluency, and completeness of generated reports using a 1-5 Likert scale. Experts also completed a Turing-like test, blindly identifying reports as human or machine-generated.

**Results:**

Phi-4 achieved the best overall performance (ROUGE-1: 0.70, BERTScore: 0.83). Expert evaluation confirmed high-quality reports in both languages (overall 4.6/5.0). Medical experts performed better than chance but struggled to differentiate human versus machine reports (accuracy: 0.60). Automatic classifiers showed strong performance (accuracy: 0.98).

**Discussion:**

The high quality of LLM-generated reports supports their potential to enhance healthcare efficiency in multilingual settings. However, the discrepancy between human detection difficulty and automated detection success reveals inherent limitations in relying solely on human oversight for quality assurance and misuse prevention.

**Conclusions:**

Deployment of LLMs for medical reporting requires combining automated detection tools with human expertise to ensure patient safety. Dataset and code: https://github.com/ds4dh/medical_report_generation.

## Background and significance

Medical documentation is essential for healthcare delivery, yet it imposes a pervasive administrative burden that reduces time for direct patient care and contributes to clinician burnout.^1–3^ Consequently, automated medical report generation has emerged as a key research priority, with the potential to streamline clinical workflows and enhance healthcare efficiency.^4–6^ Recent advances in large language models (LLMs) are disrupting this traditional process, demonstrating outstanding proficiency in capturing complex medical terminology and generating contextually appropriate clinical text to streamline workflows.^7–11^

Despite this promise, the responsible integration of LLMs for clinical documentation faces several, yet underexplored, challenges beyond the known issues of medical terminology, electronic health record (EHR) heterogeneity, and data access regulations.^12–15^ First, a gap exists in understanding model performance in English compared to other languages with different resource levels. Research has predominantly focused on English, leaving the quality and reliability of LLMs in non-English languages largely understudied.^16^ As health systems globally integrate these tools, understanding whether open-weight models can perform safely across languages, including those with moderate resources like French, is essential for equitable deployment. Despite advances in multilingual LLMs, training data remains predominantly English. For example, the corpus used to train the Phi-4 language model is composed of about 92% of English documents.^17^^,^^18^ Similarly, a recent review identified that while as many as 56% of clinical text datasets for medical AI are in English, other languages, such as French, Japanese, and Portuguese, compose only 2% of the resources.^19^^,^^20^ Second, the goal of generating human-quality text introduces a fundamental challenge for safe clinical deployment. While creating reports that are indistinguishable from those written by experts is a benchmark for success, it also might present considerable safety risks if clinicians cannot reliably identify purely generated AI outputs. We term this the *detectability paradox*: as generation quality rises, the ability of human oversight to audit the provenance of clinical documentation declines, potentially allowing hallucinations to propagate unchecked.

Our work confronts these parallel challenges by systematically evaluating both the generation quality of LLMs across English and French and the ability of clinicians to distinguish this content from human-authored work. To investigate our research questions, we designed a comprehensive, multilingual evaluation framework. The foundation of this work is BiMedReport-4K, a novel bilingual dataset we constructed of 4212 authentic medical reports in English and French, sourced from clinical transcripts and peer-reviewed case reports. Using this dataset, we benchmarked several open-weight LLMs by generating new medical reports from the synthetic EHRs. The quality of these generated reports was then assessed through a 2-step approach. We designed a controlled, pre-clinical framework to benchmark the generation and detectability of open-weight LLMs. Our evaluation combines automated metrics with a blinded Turing-like test performed by a panel of bilingual physicians and residents, specifically aiming to quantify the human detection gap and evaluate the necessity of automated auditing tools.

## Materials and methods

### BiMedReport-4K corpus creation

We create BiMedReport-4K, a comprehensive bilingual corpus in English and French of medical reports. We integrated clinical narratives from 2 complementary sources: (1) *case reports* from PubMed and (2) *medical transcriptions* from MTSamples.com. **Case report subcorpus:** For the French case report subcorpus, we retrieved a set of scientific publications from PubMed and filtered by case report type, French language, and availability in PubMed Central for the full text. Additionally, we applied temporal constraints between 2000 and 2022 to exclude potentially AI-generated content, retaining only peer-reviewed, open-access publications. We then extracted case presentation sections, resulting in 1706 French clinical narratives. For the English counterpart, we randomly sampled an equivalent number of case reports from the PMC-Patient corpus.^21^  **Medical transcript subcorpus:** To enhance domain coverage across medical report types, we supplemented the corpus with 400 English medical transcriptions randomly selected from MTSamples, stratified by specialty and document type. To create French medical transcripts, we employed neural machine translation (DeepL) on the English samples. While reviewed by bilingual medical students to ensure clinical fidelity, we classify this subset as synthetic-translated data, distinct from the native-authored French case reports, to account for potential translation artifacts in the source text.

**Figure 1.**
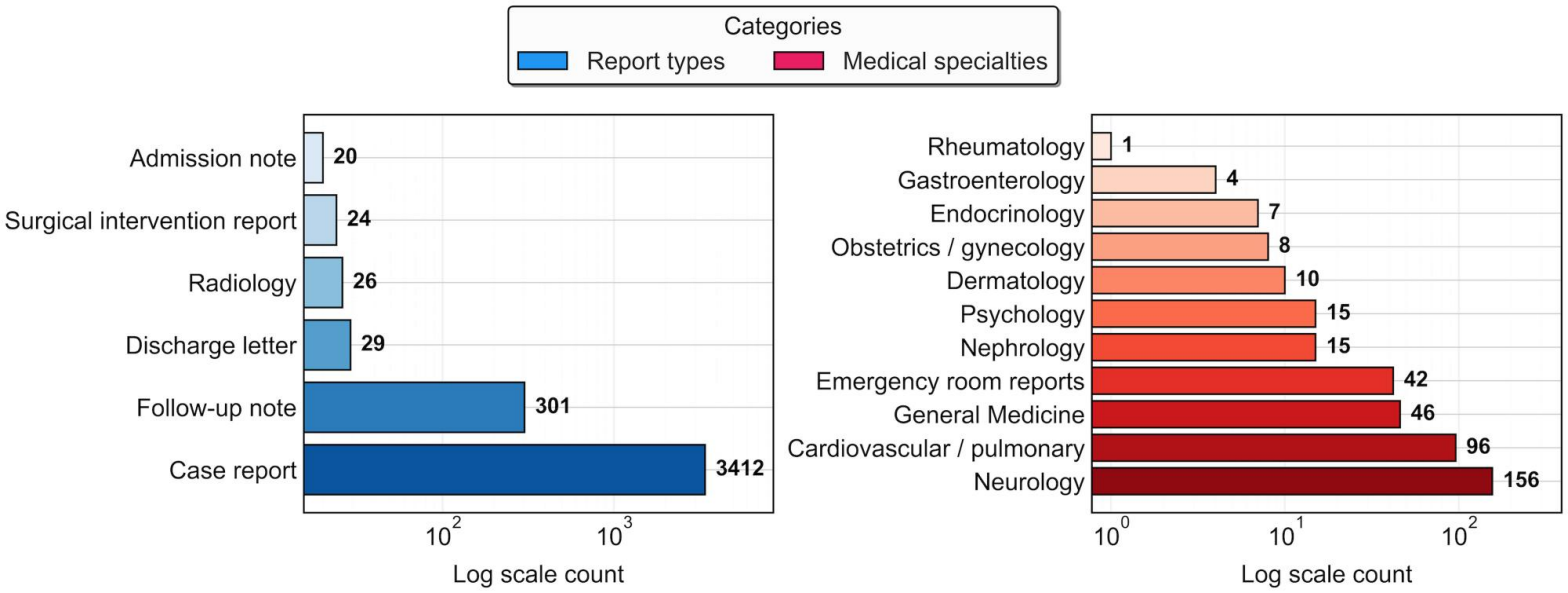
Distribution of the dataset, stratified by report type (left) and specialty (right).

We partitioned each data source—case reports and medical transcripts—separately for each language, allocating 20% to the development set and 80% to the test set. Table 1 presents general statistics of BiMedReport-4K, with detailed breakdowns of report types and specialty distributions shown in Figure 1. In total, the corpus contains 4212 medical reports, equally distributed in English and French documents, covering 6 types of medical reports—*case report*, *follow-up note*, *discharge letter*, *admission note*, *radiology report*, and *intervention report—*across 11 specialties, including neurology, cardiovascular, and general medicine.

**Table 1.** General statistics of the BiMedReport-4K dataset.

Split	Language	Source	Docs	Sentence/doc	Token/doc	Specialty	Report types
Dev	English	Case report	342	24	702	–	1
		Medical transcript	80	57	879	9	5
	French	Case report	342	16	632	–	1
		Medical transcript	80	57	1106	9	5
Test	English	Case report	1364	23	697	–	1
		Medical transcript	320	64	1052	10	5
	French	Case report	1364	16	629	–	1
		Medical transcript	320	64	1231	10	5
All			4212	28	748	11	6

### Synthetic EHR creation from medical reports via entity extraction

**Figure 2.**
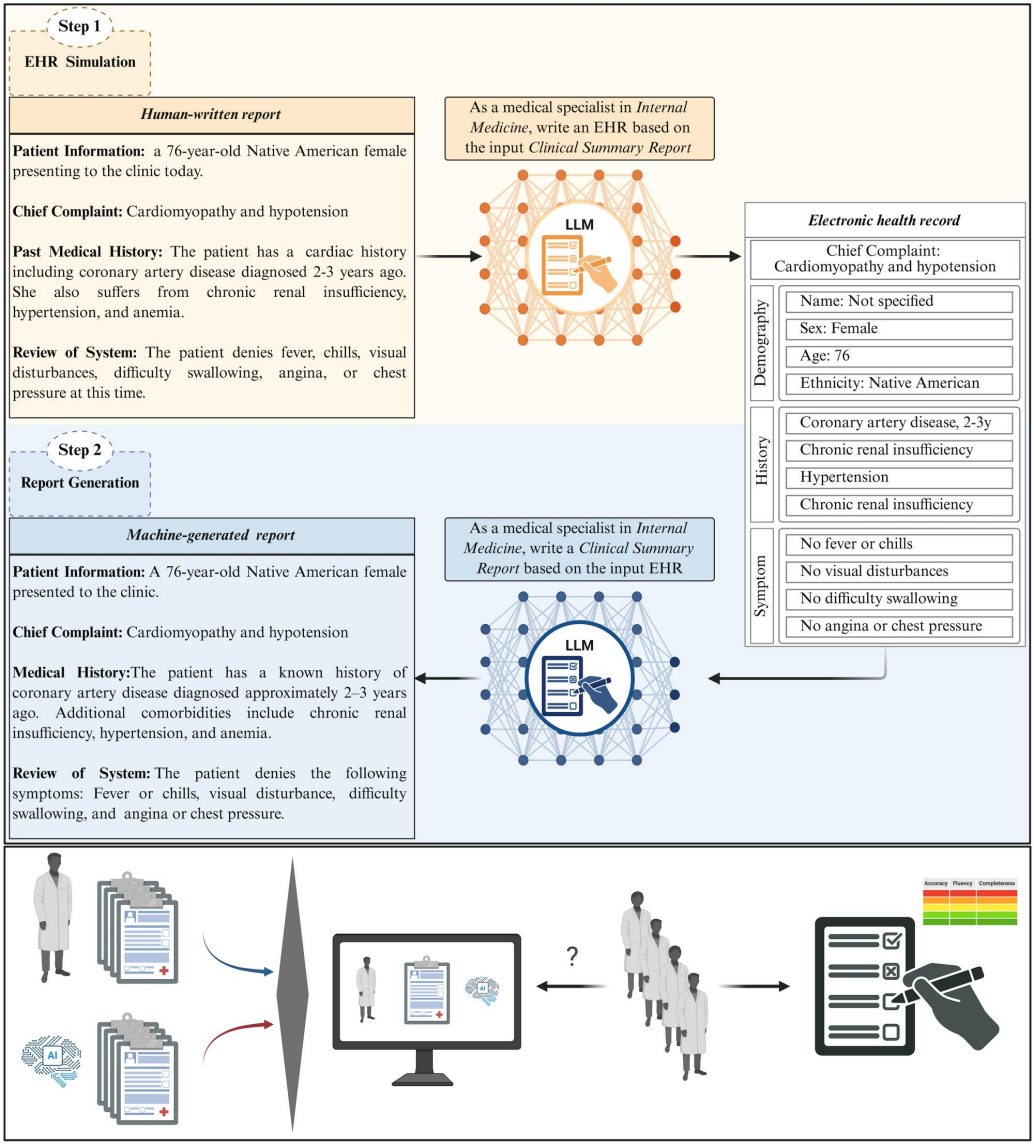
Overview of the evaluation pipeline for generating a clinical summary report by a specialist. In the first step, based on a human-written report, a synthetic, structured EHR is created using an LLM. In the second step, another LLM is used to create the same type of medical report based on the synthetic EHR.

To assess generation capabilities, we utilized a 2-stage evaluation framework (Figure 2). First, we simulated structured EHR data by extracting clinical entities from the human-authored reports using an LLM. Second, we tasked the target models with regenerating the medical report based solely on this simulated EHR data. We evaluated multiple prompting strategies, including zero-shot and few-shot (3 exemplars) configurations. See the prompt template in Figure S1.

### LLM-based medical report generation

As shown in Figure 2—Step 2, the report generation phase implements a structured-to-text conversion process that transforms the simulated EHR data into coherent, specialty-specific narratives using LLMs. We formulate this as a conditional text generation task, in which the model must synthesize clinically accurate reports while maintaining stylistic and structural fidelity to the target report type. Our generation framework takes the extracted entity set and metadata (ie, report type and specialty) as input and produces a synthetic report. We implement entity-aware generation, where extracted entities serve as semantic anchors throughout the generation process. See Figure S2 in the Supplementary for prompt details.

For LLMs equipped with reasoning capabilities during inference, specifically, GPT-OSS and the Qwen-3 family, we implemented multiple prompting configurations that allowed for the selective activation or deactivation of reasoning modes. For GPT-OSS, we tested 3 reasoning levels (low, medium, and high), whereas for Qwen-3 models, reasoning was either enabled or disabled. For each configuration, we employed 2 prompting paradigms: (1) *zero-shot*, where the model was given only task-specific instructions, and (2) *few-shot*, where the prompt included a small number of exemplars as EHR–report pairs. To determine the optimal number of exemplars, we systematically varied this parameter using Qwen-3-8B (Qwen/Qwen3-8B)^11^ as the medical report generator. We evaluated prompts including 1, 3, 5, or 10 exemplars and found that incorporating 3 examples yielded the best overall performance. The comparison results are shown in Table S1 in the Supplementary Information. These different hyper-parameter optimization experiments were conducted using the BiMedReport-4K development set. Two example reports generated by the best-performing LLM in English and French are provided in Figures S3 and S4 of the Supplementary Information, respectively.

**Figure 3.**
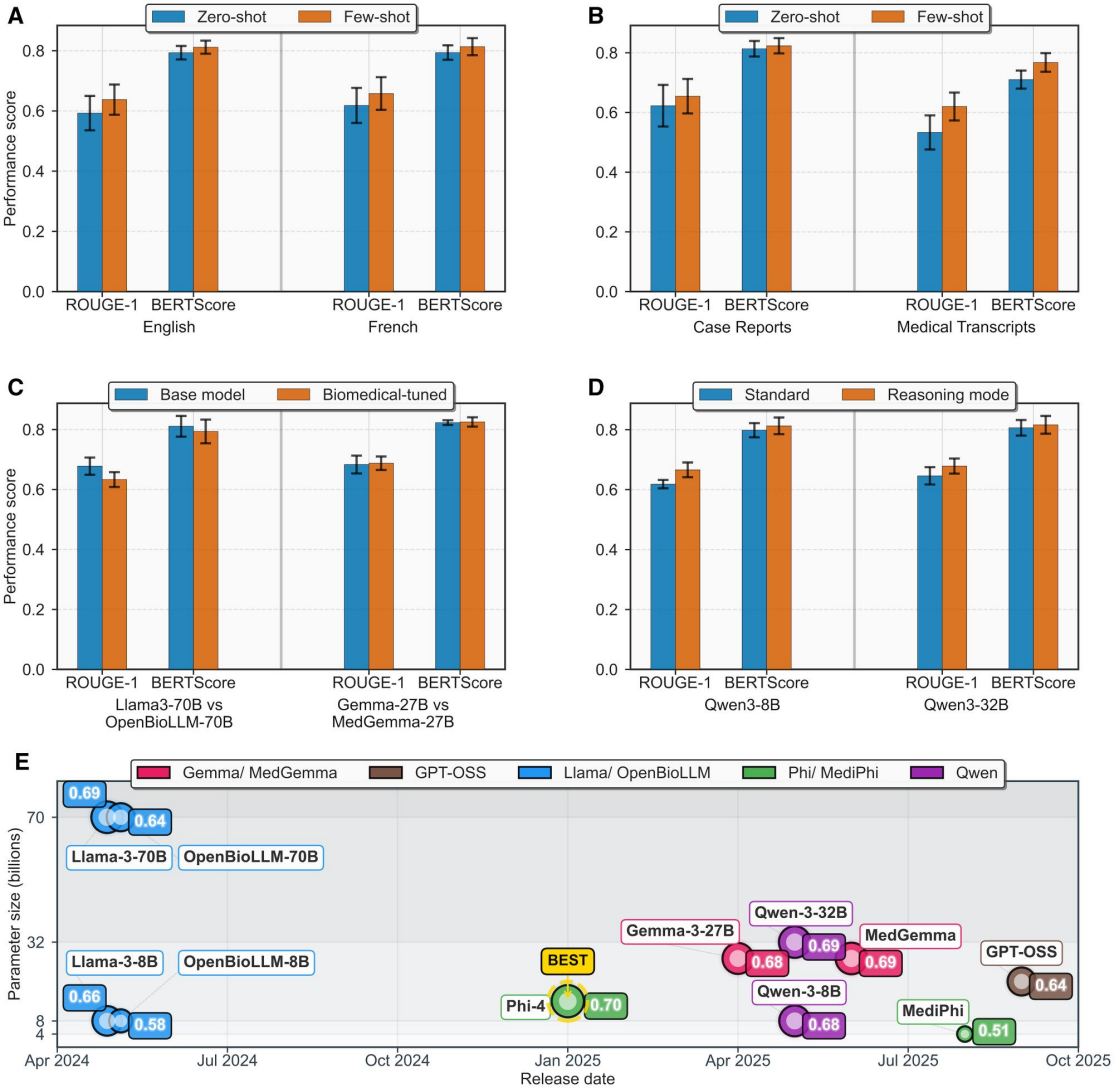
Performance comparison of LLMs on the BiMedReport-4K corpus by language (Phi-4—Panel A), report type (Phi-4—Panel B), domain pretraining (Panel C), reasoning mode (Panel D), and model size and recency (Panel E).

**Figure 4.**
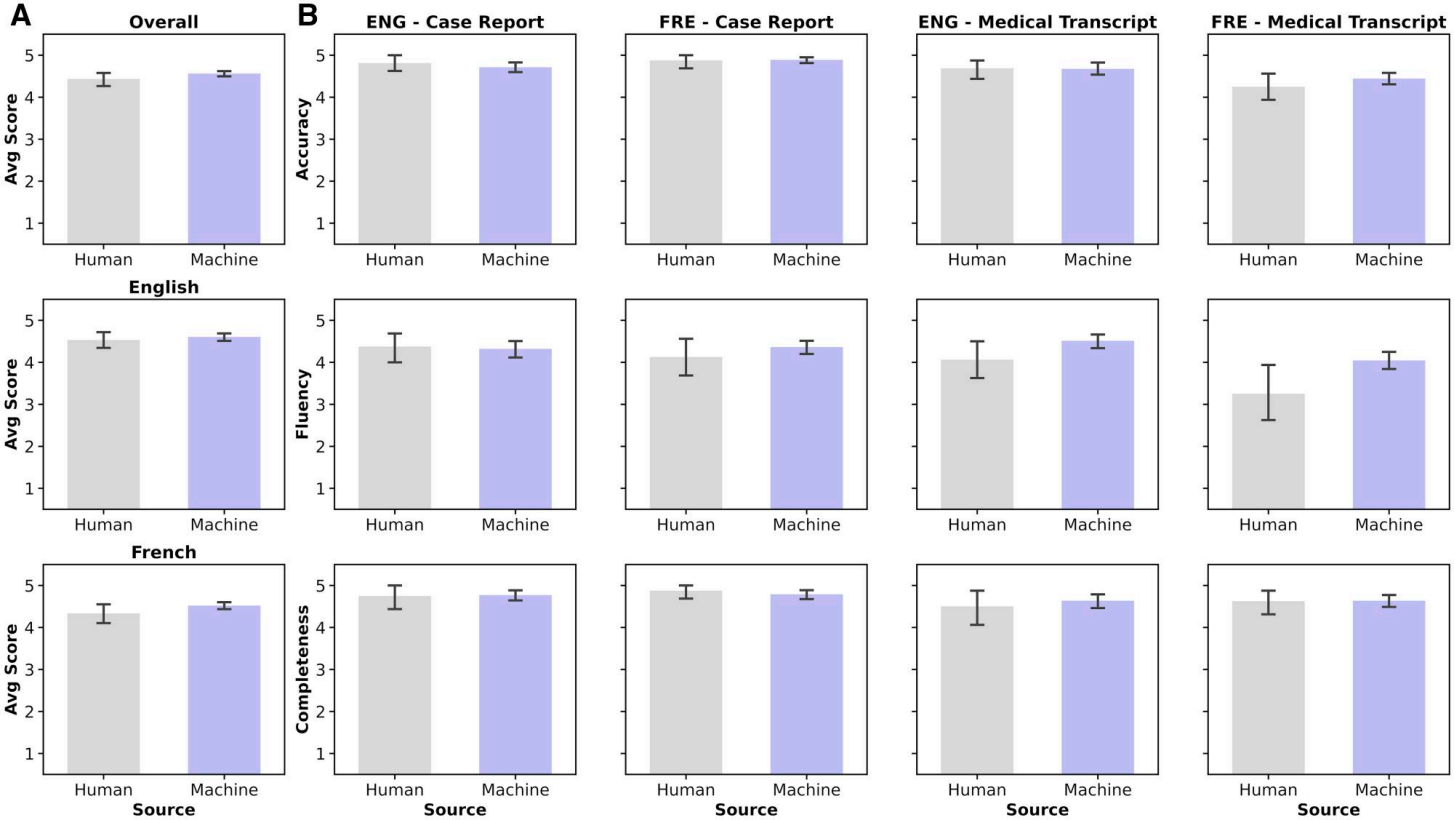
Expert quality evaluation of EHRs versus machine-generated medical reports. (A) Overall average scores across all 3 dimensions (accuracy, fluency, completeness); (B) Accuracy, fluency, and completeness scores stratified by language and document type.

## LLMs assessed for synthetic EHR creation and medical report generation

We evaluated the following general-purpose and biomedical-specialized LLMs, covering parameter sizes from 8B to 70B:

- General-purpose models: Gemma-3-27B (google/gemma-3-27b-it),^22^ GPT-OSS (openai/gpt-oss-20b),^23^ Llama-3-8B (meta-llama/Meta-Llama-3-8B-Instruct),^24^ Llama-3-70B (meta-llama/Meta-Llama-3-70B-Instruct),^24^ Phi-4 (microsoft/phi-4),^17^ Qwen-3-8B (Qwen/Qwen3-8B),^25^ and Qwen-3-32B (Qwen/Qwen3-32B).^25^
- Biomedical-specialized models: MedGemma (google/medgemma-27b-text-it),^26^ MediPhi (microsoft/MediPhi-Instruct),^27^ OpenBioLLM-8B (aaditya/Llama3-OpenBioLLM-8B),^28^ and OpenBioLLM-70B (aaditya/Llama3-OpenBioLLM-70B).^28^

Model selection was based on 3 main criteria: (1) open-weight availability, (2) multilingual capabilities, and (3) diversity in model characteristics, including parameter scale, reasoning capabilities, and training domains. These criteria are motivated by the following rationale: (1) open-weight models have better safety and privacy properties compared to cloud-based counterparts; (2) the need for assessing clinical documentation beyond English; and (3) computational resources in healthcare settings vary greatly, with many of the hospitals having low-capacity infrastructure for AI. Details for model selection for the EHR simulation step are reported in the Supplementary Information.

To benchmark open-weight performance against current industry standards, we additionally evaluated GPT-5^29^ using the same pipeline on a randomized subset of 50 reports. This allows for a direct comparison between privacy-preserving open-weight models and state-of-the-art proprietary systems.

### Qualitative evaluation of the generated reports by experts

Nine medical experts, 4 certified physicians, 5 medical students, evaluated 50 reports per language (100 total) against their corresponding EHRs using 3 criteria on a 5-point Likert scale (1 = poor, 5 = excellent): (1) accuracy, ie, consistency and correctness of clinical information, (2) fluency, ie, linguistic quality and narrative coherence, and (3) completeness, ie, thoroughness of information capture. As a control, 25% of the evaluated reports were human-written. Experts received evaluation instructions and metadata for each report, including the document category (case report or medical transcript), specific report type, and medical specialty. All experts independently scored reports without knowledge of authorship to ensure reliability and minimize bias. Detailed annotation instructions are provided in the Supplementary—Annotation instructions.

### Turing-like test with medical experts

In this experiment, the medical experts participated in a Turing-like test, classifying reports as human-authored or machine-generated without knowledge of true authorship. Nine medical experts—4 board-certified physicians and 5 master’s medical students—received evaluation instructions along with metadata for each report: category (case report/medical transcript), report type, and medical specialty. We also removed the formatting style to avoid artefacts. Each expert evaluated 50 reports per language (100 total), balanced between human-written and LLM-generated content using the Phi-4 model.

Both evaluation tasks—the qualitative assessment (Task 1) and the Turing-like authorship test (Task 2)—were distributed to the expert panel simultaneously, without a mandated completion order. Task 1 was designed to be authorship-agnostic: reports were presented in a blinded fashion without labels indicating whether they were machine-generated or human-written, and included human-authored controls.

### Machine learning-based automated text classifier

For each instance of the BiMedReport-4K corpus, we selected either the original human-written report or the machine-generated version produced by Phi-4, creating a balanced binary classification dataset of *n *= 2106 instances per language. Each language-specific dataset was stratified by report type and partitioned into training (70%, *n *= 1474), validation (15%, *n *= 316), and test (15%, *n *= 316) sets. Then, we trained a semantic-based classifier on the training set by fine-tuning EuroBERT,^30^ a multilingual transformer supporting English and French languages. The model with the highest validation accuracy on the development set was saved during training.

### Evaluation metrics and statistical analyses

We employed multiple evaluation metrics tailored to each experimental task. For report generation quality, we used ROUGE-1^31^ F1 to measure lexical overlap through unigram F1-scores and BERTScore^32^ to capture semantic similarity through contextual embeddings, both computed against human-authored reports in the BiMedReport-4K test set. Full descriptions of all evaluation metrics are available in the Supplementary Information (“Evaluation metrics explained”).

We conducted multiple statistical tests to validate experimental findings across generation quality, detection performance, and expert evaluation tasks, with all tests using a significance threshold of α = 0.05. For model comparisons, we conducted paired *t*-tests. To evaluate whether the best-performing model approached the paraphrase benchmark, we conducted a one-sample, one-sided *t*-test. Performance differences between conditions (English vs French, zero-shot vs few-shot, general-purpose vs biomedically-adapted models) were assessed using paired *t*-tests, while effects of model recency and parameter size on generation performance were evaluated using linear regression. Machine classifier accuracy was evaluated using logistic regression. Language-specific differences in expert quality ratings (accuracy, fluency, completeness) were assessed using mixed-effects models. To test whether human experts could detect machine-generated reports above chance level, we used a linear mixed-effect model.

## Results

### Evaluation pipeline

We performed all experiments on the BiMedReport-4K corpus. To assess LLM capabilities in medical report generation in both English and French, we developed a 2-stage evaluation framework (Figure 1). In Step 1—EHR Simulation, an LLM extracts structured clinical entities from human-authored medical reports to simulate EHR data. In Step 2—Report Generation, a second LLM generates medical reports from these simulated (or synthesized) EHRs. This bidirectional design enables controlled evaluation: by comparing generated reports against original human-authored reports, we quantify how accurately LLMs preserve clinical information through the extraction-generation cycle.

### Performance of LLMs for bilingual medical report generation

#### Quantitative performance of LLMs for medical report generation

In this experiment, we answer the first research question by evaluating the capability of various LLMs to generate medical reports from structured EHRs using the BiMedReport-4K corpus. Given the structured EHR data and the desired report type, models were prompted to create clinically coherent medical reports in the target language: English or French. We evaluated 2 prompting strategies: zero-shot, where LLMs rely solely on prior knowledge, and few-shot, where models receive 3 exemplar EHR-report pairs before processing the test input.

As shown in Table 2, Phi-4 (with 14 billion parameters) achieved the highest performance using few-shot prompting (ROUGE-1: 0.70; BERTScore: 0.83), with results comparable to Llama-3-70B, Qwen-3-32B, and MedGemma (27B parameters) (ROUGE-1: 0.69; BERTScore: 0.83). When benchmarked against the proprietary state-of-the-art GPT-5^29^ in a subset of 50 random records using few-shot, Phi-4 demonstrated comparable capability. GPT-5 achieved only marginally higher scores (ROUGE-1: 0.72; BERTScore: 0.83; *P* = 0.03), indicating that privacy-preserving open-weight models can approach the performance ceiling of commercial systems in this domain. Detailed results stratified by document type and language are shown in Figure S5, and an ablation study considering MedGemma as the source model for the EHR simulator step is shown in Table S2.

**Figure 5.**
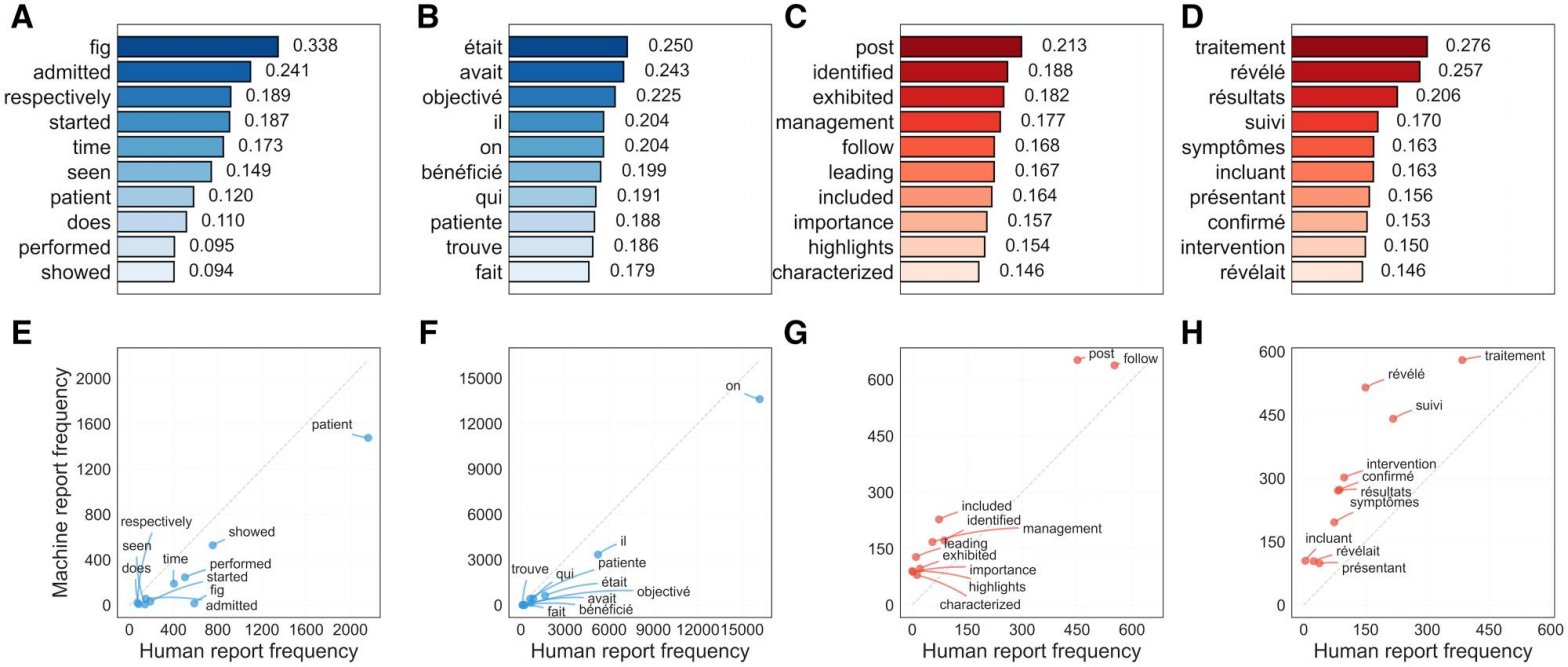
Most influential words for distinguishing authorship in medical reports. Row 1: Blue–human-written markers (A: English, B: French); Red–machine-generated markers (C: English, D: French). Row 2: Blue–human-written markers (E: English, F: French); Red–machine-generated markers (G: English, H: French).

**Table 2.** LLM performance on the BiMedReport-4K test set for different LLMs evaluated in our experiments.

LLM	ROUGE-1 score		BERTScore	
	Overall zero-shot	Overall few-shot	Overall zero-shot	Overall few-shot
Gemma-3-27B	0.64	0.68	0.81	**0.83**
MedGemma	0.62	0.69	0.78	**0.83**
GPT-OSS-low	0.61	0.64	0.79	0.80
GPT-OSS-mid	0.61	0.63	0.79	0.80
GPT-OSS-high	0.61	0.64	0.79	0.80
Llama3-8B	0.53	0.66	0.80	0.82
Llama3-70B	0.65	0.69	0.81	**0.83**
OpenBioLLM-8B	0.53	0.58	0.74	0.75
OpenBioLLM-70B	0.62	0.64	**0.82**	0.82
Phi-4	**0.68**	**0.70**	**0.82**	**0.83**
MediPhi	0.47	0.51	0.76	0.77
Qwen-3-8B	0.63	0.62	0.80	0.81
Qwen-3-8B-thinking	0.61	0.68	0.80	**0.83**
Qwen-3-32B	0.61	0.66	0.80	0.82
Qwen-3-32B-thinking	0.65	0.69	0.81	**0.83**
Min	0.47	0.51	0.74	0.75
Mean	0.61	0.65	0.79	0.81
Max	0.68	0.70	0.82	0.83

Bold values indicate the best score in each column.

As shown in Figure 3, the best open-weight LLM demonstrated comparable performance across languages (*P* < 0.001). However, document type significantly influenced quality: structured case reports yielded higher scores than heterogeneous medical transcripts (zero-shot BERTScore 0.81 vs 0.71; *P* < 0.001). Few-shot prompting substantially mitigated this gap, improving transcript generation by 8% (*P* < 0.001), demonstrating the value of exemplars for more complex clinical reports that are underrepresented in pre-training data. Biomedical-adapted LLMs did not consistently outperform general-purpose counterparts (*P* < 0.001). Enabling reasoning mode improved performance (*P* < 0.001), with ROUGE-1 increases of 2-4% and BERTScore improvements of approximately 1%. Neither parameter count (*P* = 0.32) nor release date (*P* = 0.79) guaranteed superior performance. Phi-4 (14B parameters, mid-2025) slightly outperformed larger models, including Llama-3-70B (70B) and Qwen-3-32B (32B), as well as more recent releases such as Gemma-3-27B.

#### Qualitative evaluation of the generated reports by experts

Quality evaluation by 9 medical experts in 100 medical reports resulted in most scores exceeding 4.0/5.0 in the 5-point Likert scale, with accuracy ranging from 4.4 to 4.9, fluency from 4.0 to 4.5, and completeness from 4.6 to 4.8. We observed a performance divergence based on the source of the French data. Natively authored French case reports achieved near-perfect accuracy (4.9/5.0). In contrast, machine-translated French transcripts scored significantly lower in fluency (4.0/5.0) compared to their English counterparts (4.5/5.0). This suggests that the lower resource performance drop is likely to stem from translation artifacts in the source training data rather than a limitation of the LLM’s French generation capabilities. Completeness remained consistently high across all conditions (4.6-4.8), with minimal variation between document types and languages. Overall, LLM-generated reports demonstrated strong clinical accuracy and information completeness across the different report types evaluated. Figure 4 illustrates the quality assessment stratified by evaluation dimension (accuracy, fluency, completeness), language (English, French), and document type (case reports, medical transcripts). A prototype of the evaluation tool is available here: https://heg-rl001.hesge.ch/clinicaldoceval/.

#### Distinguishability between human- and machine-generated medical reports

In this experiment, we assess whether LLM-generated medical reports can be distinguished from human-authored reports. We conducted 2 complementary evaluations: (1) a Turing-like test with medical experts, and (2) an automated text classification based on machine learning.

#### Turing-like test with medical experts

Detection accuracy by medical experts remained near chance level across all conditions (Table 3), although annotators performed slightly above chance level in identifying the origin of the reports (*P* = 0.04). Overall accuracy ranged from 58% to 61% (0.60 per average) across document types and languages. Stratified analysis revealed no differences between English and French (0.59, *P* = 0.70). Additionally, experts demonstrated marginally better performance in distinguishing authorship for medical transcripts compared to case reports (0.60-0.61 vs 0.58-0.59; *P* = 0.80), indicating that as report quality improves, human verification becomes an increasingly unreliable safety control.

**Table 3.** Medical experts’ accuracy in distinguishing human-authored from machine-generated medical reports in a Turing-like evaluation.

Report type (Language)/Accuracy	Case reports (ENG)	Case reports (FRE)	Medical transcripts (ENG)	Medical transcripts (FRE)	Average (ENG)	Average (FRE)	Average (All)
Human	0.58	0.59	0.61	0.60	0.59	0.59	0.60
Machine	0.98	0.97	0.98	0.98	0.98	0.98	0.98

#### Machine learning-based automated text classifier for LLM-generated content

As shown in Table 3, in contrast to human experts, the machine learning-based classifier leveraging EuroBERT^18^ achieved a robust detection performance with an overall accuracy of 0.98 (*P* < 0.001). This validates the detectability paradox: while LLMs produce clinically convincing text, they leave a distinct digital fingerprint, characterized by specific lexical patterns (Figure 5) that semantic classifiers can reliably identify. This suggests that automated auditing is a viable and necessary alternative to human oversight for tracking AI provenance.

To try to identify potential patterns that could be exploited by the machine learning model to distinguish human—from LLM-generated content, we considered 2 linguistic features—vocabulary and part-of-speech—as they have been shown to diverge from human-written text,^33^ in addition to de-identification artifacts. To identify vocabulary features, we computed the most influential words impacting the decision of the classification model for both English and French sets using integrated gradient scores.^22^ Word-level scores were computed by averaging subword token scores. Figure 5A–D displays the top 10 most influential words for each authorship class, stratified by classifier, authorship (human in blue, machine in red), and language, revealing distinct lexical patterns characteristic of machine-generated content. As shown in Figure 5E–H, these words tend to appear more frequently in one or another corpus. While individually they cannot discriminate the generation source, we hypothesize that their aggregated score allows the machine learning to discriminate between human and machine-generated documents. An analysis for the first 1000 most relevant words is provided in Figure S6. For the part-of-speech features, we retrained the model on reports with all punctuation removed. The results show an impact on accuracy of -2%, which highlights other subtle factors that help the model to differentiate the authorship source. Finally, for the de-identification artifacts, we compared the performance on case reports, which are clean of de-identification artifacts, with medical transcripts, which contain passages such as “abc *who is 10 years of age*” or “*reviewed prior to this conference by* x, rn, bsn.” Results show that the difference between case reports and medical transcripts is negligible (< 0.1%).

## Discussion

Our study identifies an important detectability paradox in the deployment of generative AI for clinical documentation. While open-weight LLMs demonstrated the capability to generate bilingual medical reports of near-human quality (rated 4.6/5.0 by experts), this fidelity creates a challenge: clinical experts were unable to reliably distinguish AI-generated content from human-authored text (60% accuracy). This finding challenges the prevailing assumption that human-in-the-loop oversight is sufficient to validate the provenance of clinical documentation. However, our results suggest a technical solution to this safety gap. Despite bypassing human detection, LLM-generated reports retained a distinct digital fingerprint, characterized by specific lexical patterns, that allowed an automated classifier to detect them with 98% accuracy. This discrepancy implies that health systems cannot rely solely on clinician vigilance to detect AI use. Instead, the integration of automated auditing tools or watermarking classifiers must be considered a prerequisite for safe deployment to prevent the unchecked propagation of AI-generated errors.

Our findings challenge the assumption that high-quality clinical generation requires massive proprietary models or English-only resources. The open-weight model Phi-4 (14B parameters) matched the performance of larger models (70B) and demonstrated robust cross-lingual transfer, achieving comparable quality in French despite the vast disparity in training data availability. Additionally, general-purpose models consistently outperformed biomedical-specialized counterparts (eg, MediPhi, OpenBioLLM). This suggests that for clinical documentation, the reasoning and linguistic capabilities acquired during large-scale pretraining are more valuable than domain-specific fine-tuning,^34^ making efficient, open-weight models a viable solution for diverse healthcare settings.

However, generation quality is heavily dependent on prompting strategies and data provenance. We observed that few-shot prompting (providing 3 exemplars) was beneficial for routine medical transcripts, yielding a 9% performance gain compared to zero-shot approaches. This improvement was less pronounced for standardized case reports, likely due to the model’s exposure to CARE guidelines during pretraining.^35^ Additionally, the performance divergence between natively authored French reports (high accuracy) and machine-translated transcripts (lower fluency) indicates that low-resource limitations may often stem from the quality of evaluation datasets rather than the model’s intrinsic linguistic capabilities.

Our study presents several limitations. The in-silico evaluation relies on synthetic EHRs, which may not capture the temporal complexity of raw clinical data. Moreover, human-authored references (peer-reviewed case reports and curated transcriptions) are more polished than authentic hospital documentation, which typically features telegraphic syntax and informal shorthand^36^—narrowing the stylistic gap and making AI authorship harder to detect. Our findings, therefore, represent a conservative estimate of real-world detectability. Nevertheless, expert detection remained near chance (0.58-0.61), indicating that clinicians could not reliably identify AI-generated content within this reduced gap. Validating these findings on authentic, de-identified clinical notes remains an important future direction. Additionally, reliance on machine-translated French transcripts limits validity for that document type, necessitating future work with natively authored corpora. Finally, while the expert panel is relatively small (*n *= 9), strong alignment between automated classifiers and near-chance detection rates supports the robustness of our findings. Further limitations relate to our methodology and corpus. First, experts completing the unmandated qualitative assessment before authorship classification may have developed implicit sensitivity to AI stylistic patterns, despite the absence of labels and inclusion of human controls. The 60% detection accuracy thus likely represents an upper bound; real-world detection would be lower, underscoring the need for automated auditing. Second, aggregate Likert scores do not differentiate errors by clinical severity; a moderate score could reflect minor inaccuracies or dangerous omissions. Future work must quantify safety-critical failures using a granular taxonomy of patient harm to safely translate these models into practice. Finally, no French case reports were explicitly indexed with rare disease-related MeSH terms. While specific disease indexing without the overarching “Rare Diseases” heading might modestly underestimate this proportion, the scarcity of open-access French literature in this domain remains a notable limitation.

In conclusion, this study demonstrates that open-weight LLMs can generate bilingual medical reports of a quality that is indistinguishable from human-authored text. While this capability promises to alleviate administrative burdens in diverse healthcare settings, it creates a critical safety gap: human oversight alone is no longer a reliable safeguard against AI errors or misuse. As health systems integrate these technologies, the deployment of automated auditing tools, capable of detecting the digital fingerprint that human experts miss, must be considered a mandatory component of clinical AI governance.

## Supplementary Material

ocag070_Supplementary_Data

## Data Availability

The BiMedReport-4K dataset and the codes used to conduct the experiments are publicly available at https://github.com/ds4dh/medical_report_generation

## References

[1] Xiao H , Zhou F, Liu X, et al A comprehensive survey of large language models and multimodal large language models in medicine. Inform Fusion. 2025;117:102888.

[2] Lin SY , Shanafelt TD, Asch SM. Reimagining clinical documentation with artificial intelligence. Mayo Clin Proc. 2018;93:563-565. 29631808 10.1016/j.mayocp.2018.02.016

[3] Liu F , You C, Wu X, et al Auto-encoding knowledge graph for unsupervised medical report generation. Adv Neural Inf Process Syst. 2021;34:16266-16279.

[4] Zhou Y , Wang H. Divide and conquer radiology report generation via observation level fine-grained pretraining and prompt tuning. In: Al-Onaizan Y, Bansal M, Chen Y-N, eds. *Proceedings of the 2024 Conference on Empirical Methods in Natural Language Processing*. Association for Computational Linguistics, 2024:7597-7610. 10.18653/v1/2024.emnlp-main.433

[5] Yuan D , Rastogi E, Naik G, et al A continued pretrained LLM approach for automatic medical note generation. In: *Proceedings of the 2024 Conference of the North American Chapter of the Association for Computational Linguistics: Human Language Technologies* (Volume 2: Short Papers); 2024:565-571.

[6] Jung H , Kim Y, Choi H, et al Enhancing clinical efficiency through LLM: discharge note generation for cardiac patients. *arXiv* [Preprint]. 2024. Available from: https://arxiv.org/abs/2404.05144

[7] Wu X, Yang S, Qiu Z, et al. DeltaNet: conditional medical report generation for COVID-19 diagnosis. In: *Proceedings of the 29th International Conference on Computational Linguistics*; 2022:2952–2961.

[8] Azmat M, Abbas M, de Macedo MMG, et al. MEAL: A multi-dimensional evaluation of alignment techniques for LLMs. Presented at: NeurIPS 2025 Workshop on Evaluating the Evolving LLM Lifecycle; 2025. Available from: https://arxiv.org/abs/2508.09937

[9] Zhu Y , Yang X, Wu Y, Zhang W. Leveraging summary guidance on medical report summarization. IEEE J Biomed Health Inform. 2023;27:5066-5075. 37566507 10.1109/JBHI.2023.3304376

[10] Shing H-C, Shivade C, Pourdamghani N, et al. Towards clinical encounter summarization: learning to compose discharge summaries from prior notes. arXiv [Preprint]. 2021. Available from: https://arxiv.org/abs/2104.13498

[11] Gostin LO , Levit LA, Nass SJ. Beyond the HIPAA privacy rule: enhancing privacy, improving health through research. Washington, DC: National Academies Press; 2009. 20662116

[12] Rouhizadeh H , Yazdani A, Zhang B, et al Large language models struggle to encode medical concepts—a multilingual benchmarking and comparative analysis. 2025.01.15.25320579. 2025. 10.1101/2025.01.15.25320579.

[13] Hong N , Wen A, Shen F, et al Integrating structured and unstructured EHR data using an FHIR-based type system: a case study with medication data. AMIA Jt Summits Transl Sci Proc. 2018;2017:74-83. 29888045 PMC5961797

[14] Chico V. The impact of the general data protection regulation on health research. Br Med Bull. 2018;128:109-118. 30445448 10.1093/bmb/ldy038

[15] Jonnagaddala J , Wong ZS-Y. Privacy preserving strategies for electronic health records in the era of large language models. NPJ Digit Med. 2025;8:34. 39820020 10.1038/s41746-025-01429-0 PMC11739470

[16] Rouhizadeh H , Yazdani A, Zhang B, Teodoro D. Exploring zero-shot cross-lingual biomedical concept normalization via large language models. Stud Health Technol Inform. 2025;327:788-792. 40380575 10.3233/SHTI250467

[17] Abdin M, Aneja J, Behl H, et al. Phi-4 technical report. arXiv [Preprint]. 2024. Available from: https://arxiv.org/abs/2412.08905

[18] Rouhizadeh H , Nikishina I, Yazdani A, et al A dataset for evaluating contextualized representation of biomedical concepts in language models. Sci Data. 2024;11:455. 38704422 10.1038/s41597-024-03317-w PMC11069517

[19] Wu J , Liu X, Li M, et al Clinical text datasets for medical artificial intelligence and large language models—a systematic review. NEJM AI. 2024;1:AIra2400012.

[20] Xuan W , Yang R, Qi H, et al MMLU-ProX: a multilingual benchmark for advanced large language model evaluation. In: Christodoulopoulos C, Chakraborty T, Rose C, eds. *Proceedings of the 2025 Conference on Empirical Methods in Natural Language Processing*. Association for Computational Linguistics; 2025:1513-1532. 10.18653/v1/2025.emnlp-main.79

[21] Zhao Z , Jin Q, Chen F, Peng T, Yu S. A large-scale dataset of patient summaries for retrieval-based clinical decision support systems. Sci Data. 2023;10:909. 38110415 10.1038/s41597-023-02814-8 PMC10728216

[22] Gemma Team. Gemma 3 technical report. arXiv [Preprint]. 2025. Available from: https://arxiv.org/abs/2503.19786

[23] OpenAI. gpt-oss-120b & gpt-oss-20b model card. arXiv [Preprint]. 2025. Available from: https://arxiv.org/abs/2508.10925

[24] AI@Meta. Llama 3 Model Card. 2024. https://github.com/meta-llama/llama3/blob/main/MODEL_CARD.md.

[25] Yang A, Li A, Yang B, et al. Qwen3 technical report. arXiv [Preprint]. 2025. Available from: https://arxiv.org/abs/2505.09388

[26] Sellergren A, Kazemzadeh S, Jaroensri T, et al. MedGemma technical report. arXiv [Preprint]. 2025. Available from: https://arxiv.org/abs/2507.05201

[27] microsoft/MediPhi Hugging Face. 2024. https://huggingface.co/microsoft/MediPhi.

[28] Ankit Pal MS. OpenBioLLMs: advancing open-source large language models for healthcare and life sciences. Hugging Face Repository; 2024.

[29] Achiam J, Adler S, Agarwal S, et al. GPT-4 technical report. arXiv [Preprint]. 2023. Available from: https://arxiv.org/abs/2303.08774

[30] Boizard N, Gisserot-Boukhlef H, Alves DM, et al. EuroBERT: scaling multilingual encoders for European languages. arXiv [Preprint]. 2025. Available from: https://arxiv.org/abs/2503.05500

[31] Lin C-Y. ROUGE: a package for automatic evaluation of summaries. In: Text Summarization Branches Out. Association for Computational Linguistics; 2004:74-81.

[32] Zhang T, Kishore V, Wu F, Weinberger KQ, Artzi Y. BERTScore: evaluating text generation with BERT. In: *Proceedings of the 8th International Conference on Learning Representations*; 2020.

[33] Kobak D, González-Márquez R, Horvát EÁ, Lause J. Delving into LLM-assisted writing in biomedical publications through excess vocabulary. Sci Adv. 2025;11(27):eadt3813. 10.1126/sciadv.adt3813 PMC12219543 40601754

[34] Dorfner FJ , Dada A, Busch F, et al Evaluating the effectiveness of biomedical fine-tuning for large language models on clinical tasks. J Am Med Inform Assoc. 2025;32:1015-1024. 40190132 10.1093/jamia/ocaf045 PMC12089759

[35] Gagnier JJ , Kienle G, Altman DG, et al The CARE guidelines: consensus-based clinical case reporting guideline development*. Glob Adv Health Med. 2013;2:38-43. 10.7453/gahmj.2013.008 PMC3833570 24416692

[36] Soni S , Demner-Fushman D. Toward relieving clinician burden by automatically generating progress notes using interim hospital data. AMIA Annu Symp Proc. 2024;2024:1059-1068. 40417482 PMC12099345

