# Supplementary material for: The detectability paradox: bilingual medical report generation with open-weight models and the limits of human oversight
Source: J Am Med Inform Assoc. 2026 May 8;33(7):1303–13. doi: 10.1093/jamia/ocag070 (PMC13317965; doi:10.1093/jamia/ocag070)
Supplement: ocag070_Supplementary_Data [file ocag070_supplementary_data.zip › Main_Supplementary.docx]

Supplementary Information

Figure A.1: Pseudo-code for prompting for generating the synthetic EHR.

Figure A.2: Pseudo-code for few-shot prompting in the medical report generation task.

| Number of exemplars | ROUGE-1 | BERTScore |
| --- | --- | --- |
| 1 | 0.67 | 0.83 |
| 3 | **0.69** | **0.84** |
| 5 | 0.67 | **0.84** |
| 10 | 0.68 | **0.84** |

Table A.1. Performance of Qwen-3-8B with few-shot prompting on report generation using different numbers of exemplars in the prompt. Results are obtained using the development set of the BiMedReport corpus.

Figure A.3: Example of a machine-generated admission note in English produced by Phi-4.

Figure A.4: Example of a machine-generated case report in French produced by Phi-4.

# **Annotation instructions**

This section provides instructions for annotators in the qualitative assessment tasks of medical report generation: (1) Human-Machine Discrimination, and (2) Quality Assessment.

Task 1: Human-Machine Discrimination

The medical reports need to be classified as human-written (1) or machine-generated (0). These reports include medical case reports from PubMed peer-reviewed papers and medical transcripts from various specialties. For each instance, you will be provided with the following information:

- Source: PubMed or medical transcript
- Medical specialty: e.g., cardiology, neurology, oncology
- Report type: e.g., case report, discharge summary, consultation note
- Instruction: Please read each report completely and enter the label (1 or 0) in the Classification column of the evaluation file. Classify each medical report as human-written (1) or machine-generated (0). Add comments if necessary

Task 2: Quality Assessment

You will evaluate medical reports (which may be human or machine-generated) against their corresponding electronic health records (EHRs). Your task is to assess how accurately, fluently, and completely the medical report reflects the information contained in the EHR. For each instance, you will be provided with the following information:

- EHR
- Medical report
- Source (PubMed or medical transcript)
- Medical specialty (e.g., cardiology, neurology, oncology)
- Report type (e.g., case report, discharge summary, consultation note)

Evaluation process:

- Thoroughly review the EHR to understand the patient’s condition, history, and relevant
- medical data.
- Examine the medical report that claims to be based on this EHR.
- Compare the report against the EHR, focusing on accuracy, fluency, and completeness.
- Assign scores (1–5) for each criterion based on the standards below.
- Enter your score for each criterion in the corresponding column of the evaluation form.

Evaluation criteria and scoring standards:

In the following, we present the criteria used by medical experts to evaluate human versus machine report generation. To maintain conciseness, descriptions for each point scale have been omitted.

Accuracy (1–5)

- 5 points (Very accurate)
- 4 points (Accurate)
- 3 points (Moderately accurate)
- 2 points (Not very accurate)
- 1 point (Inaccurate)

Fluency (1–5)

- 5 points (Very fluent)
- 4 points (Fluent)
- 3 points (Moderately fluent)
- 2 points (Not very fluent)
- 1 point (Not fluent)

Completeness (1–5)

- 5 points (Very complete)
- 4 points (Complete)
- 3 points (Moderately complete)
- 2 points (Not very complete)
- 1 point (Incomplete)

**Machine learning based authorship classification - Hyperparameters**

We used the EuroBERT model with a 2048 token maximum sequence length, trained over 20 epochs using a batch size of 16. We employed differential learning rates (2e-6 for BERT parameters, 2e-5 for the classification layer) with the AdamW optimizer.

**Evaluation metrics explained**

**ROUGE-1** (Recall-Oriented Understudy for Gisting Evaluation): This metric measures the exact word-for-word overlap between the AI-generated report and a human-written "gold standard" reference. In a clinical context, a high ROUGE-1 score indicates that the AI successfully used the exact same vocabulary and terminology as the human clinician.

**BERTScore**: While ROUGE-1 requires exact word matches, BERTScore evaluates the underlying meaning (semantic similarity) between the AI-generated and human-written text. It uses contextual language modelling to recognise that phrases like "dyspnea" and "shortness of breath," or "hypertension" and "high blood pressure" convey the same clinical concept even if the specific words differ. A high BERTScore indicates that the AI captured the correct clinical narrative and context, regardless of phrasing variations.

To illustrate the distinction, consider the following pair of sentences:

- Reference (human-written): "The patient presents with *dyspnea* and *elevated blood pressure*."
- AI-generated: "The patient exhibits *shortness of breath* and *hypertension*."

ROUGE-1 would only credit the words shared verbatim between the two sentences — "the," "patient," and "and" — resulting in a low score, because clinically important terms like "dyspnea" versus "shortness of breath" are treated as mismatches. BERTScore, by contrast, would recognise that "dyspnea" and "shortness of breath," as well as "elevated blood pressure" and "hypertension," carry equivalent clinical meaning, and would therefore assign a much higher similarity score.

Figure A.5: LLM performance on the BiMedReport-4K test set for different LLMs evaluated in our experiments.

**Effect of the EHR simulator**

To assess the impact of EHR simulator quality on report generation performance, we conducted an ablation study comparing the best-performing EHR simulator (Phi-4) against the second-best LLM (MedGemma) identified on the development set. We evaluated report generation using three top-performing LLMs (Phi-4, Qwen-3-32B-thinking, and MedGemma) with few-shot prompting on the test set. As shown in Table A.1, Phi-4 achieved the highest report generation in both settings (ROUGE-1: 0.70 with Phi-4 EHRs and 0.66 with MedGemma EHR simulator), which is indicative of the solid capabilities of this LLM in medical report generation. Results also demonstrate that EHR simulator quality impacts generation performance. When MedGemma simulated EHRs, all report generators showed decreased performance compared to Phi-4-simulated EHRs, with ROUGE-1 scores dropping by 4-8% across models (MedGemma simulator: 0.61-0.66 vs. Phi-4 simulator: 0.69-0.70).

| EHR simulator | ROUGE-1 | | | BERTScore | | |
| --- | --- | --- | --- | --- | --- | --- |
|  | Phi4 | Qwen-3-32B-thinking | MedGemma | Phi4 | Qwen-3-32B-thinking | MedGemma |
| MedGemma | 0.66 | 0.65 | 0.61 | 0.82 | 0.81 | 0.81 |
| Phi4 | 0.70 | 0.69 | 0.69 | 0.83 | 0.83 | 0.83 |

Table A.2. Ablation study comparing EHR simulator impact on report generation performance.

Figure A.6: Convergence of word distinctiveness by frequency rank. This split violin plot illustrates the distribution of distinctiveness scores for top words used by machines (red, left) versus humans (blue, right) documents. Words are grouped into logarithmically spaced bins based on their frequency rank (y-axis), with the most frequent words (ranks 1–10) at the bottom. The distinctiveness score (x-axis) ranges from -1 (exclusively machine) to +1 (exclusively human). The dashed lines represent the median score for each group. The plot reveals a funnel effect: the highest-ranked words are polarized and specific to their source, whereas lower-ranked words (rank > 300) display lower distinctiveness, converging toward a neutral score of zero.
