# Supplementary figures and images for: The detectability paradox: bilingual medical report generation with open-weight models and the limits of human oversight
Source: J Am Med Inform Assoc. 2026 May 8;33(7):1303–13. doi: 10.1093/jamia/ocag070 (PMC13317965; doi:10.1093/jamia/ocag070)

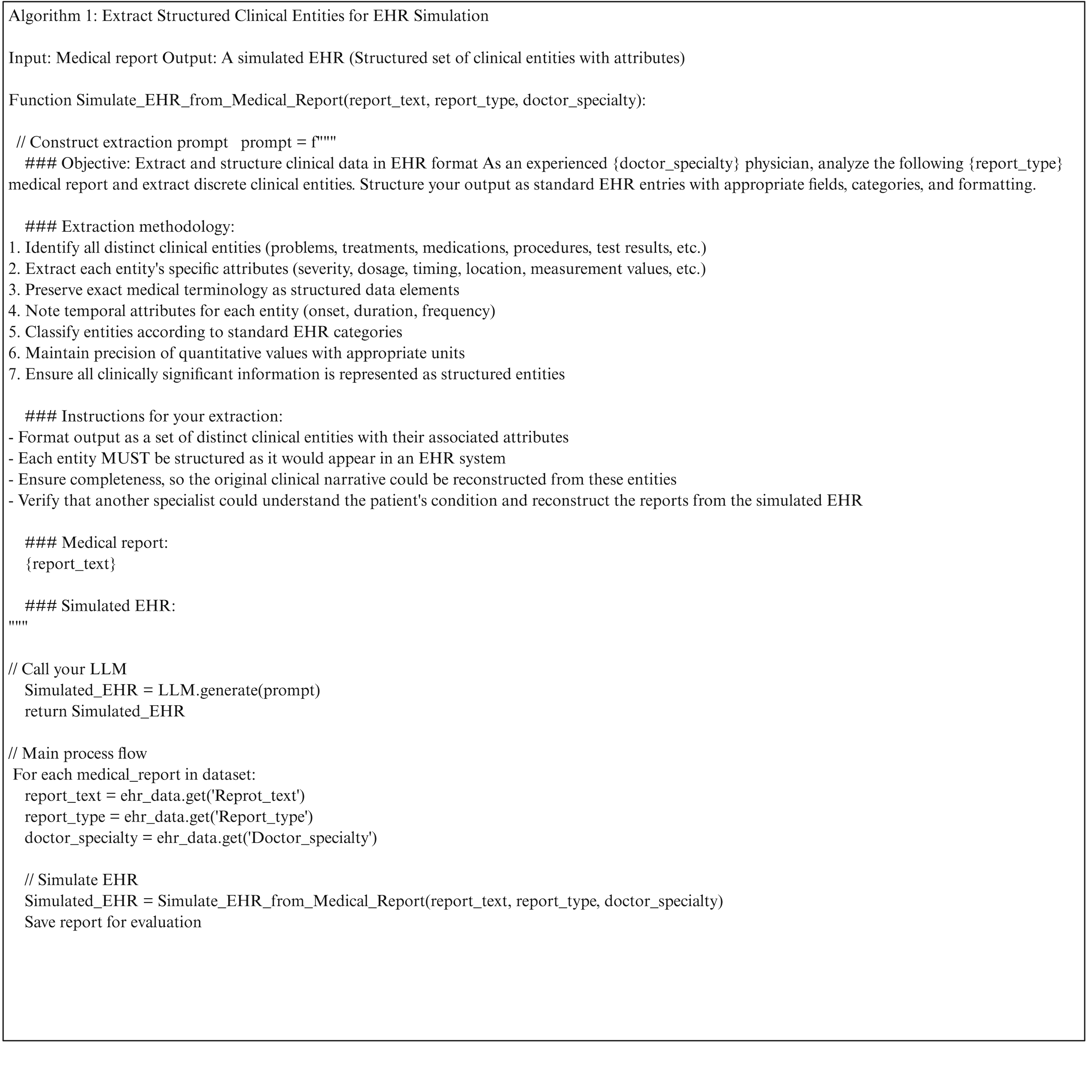

Supplement: ocag070_Supplementary_Data [file ocag070_supplementary_data.zip › Figure A1.png]

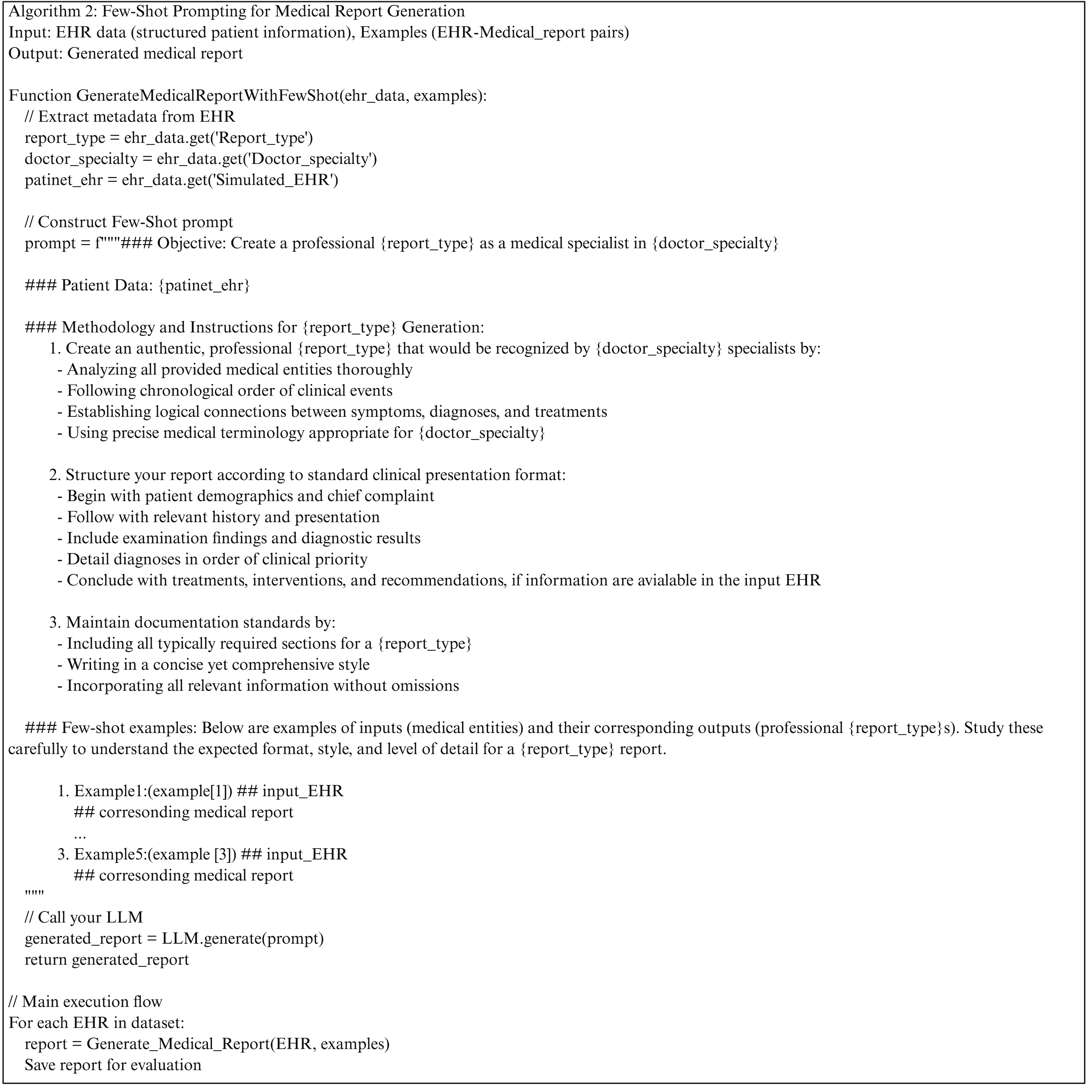

Supplement: ocag070_Supplementary_Data [file ocag070_supplementary_data.zip › Figure A2.png]

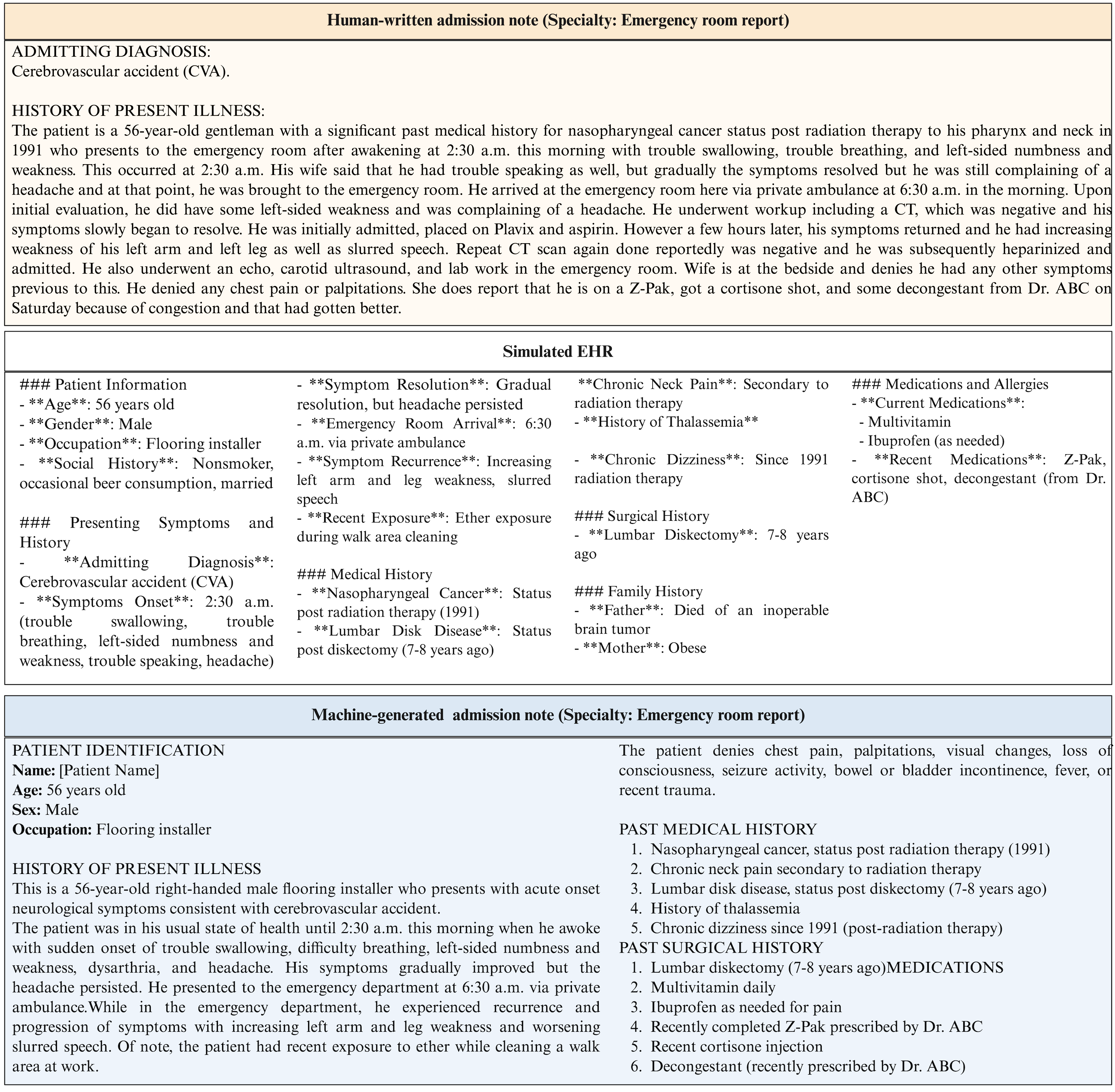

Supplement: ocag070_Supplementary_Data [file ocag070_supplementary_data.zip › Figure A3.png]

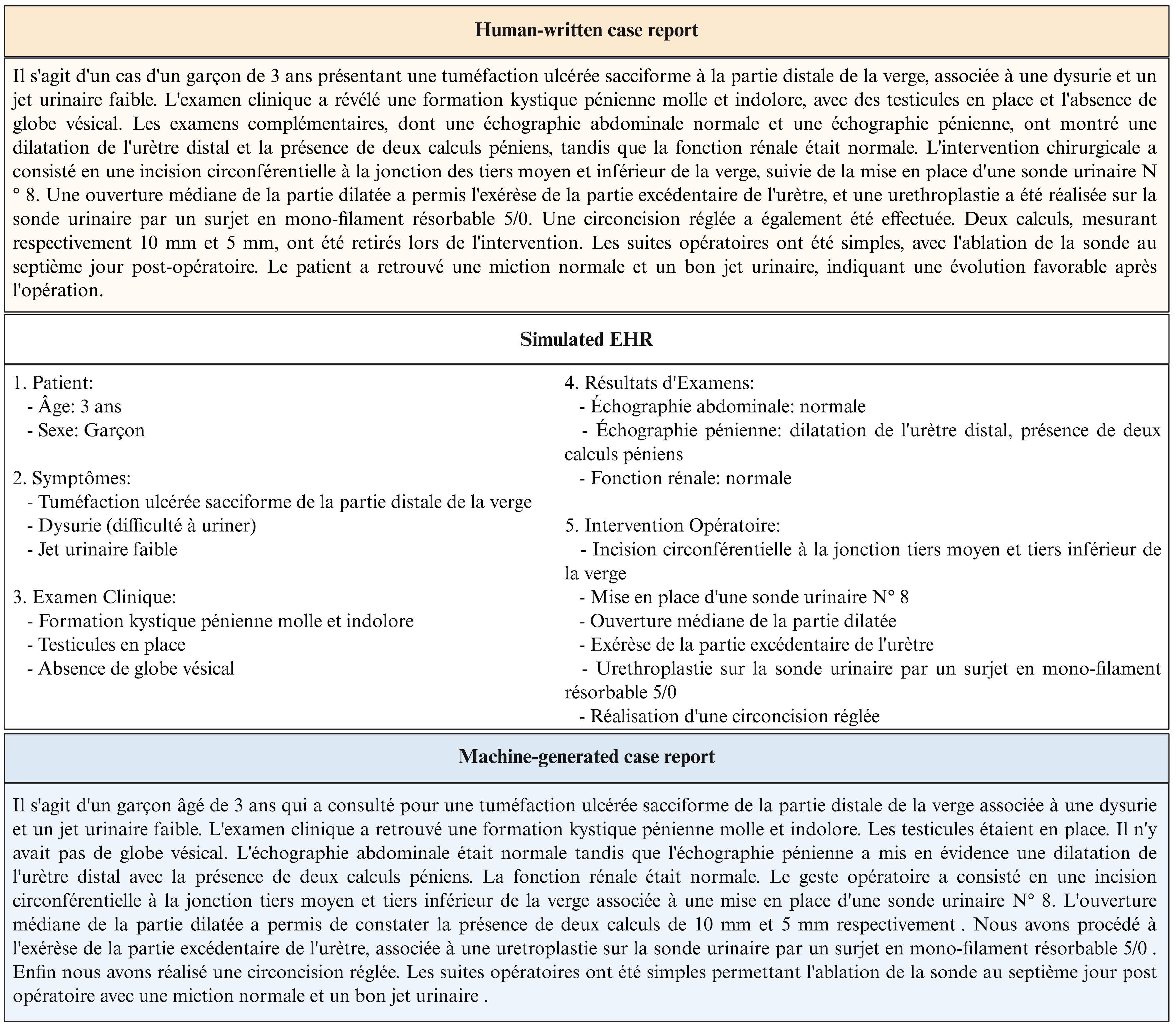

Supplement: ocag070_Supplementary_Data [file ocag070_supplementary_data.zip › Figure A4.png]

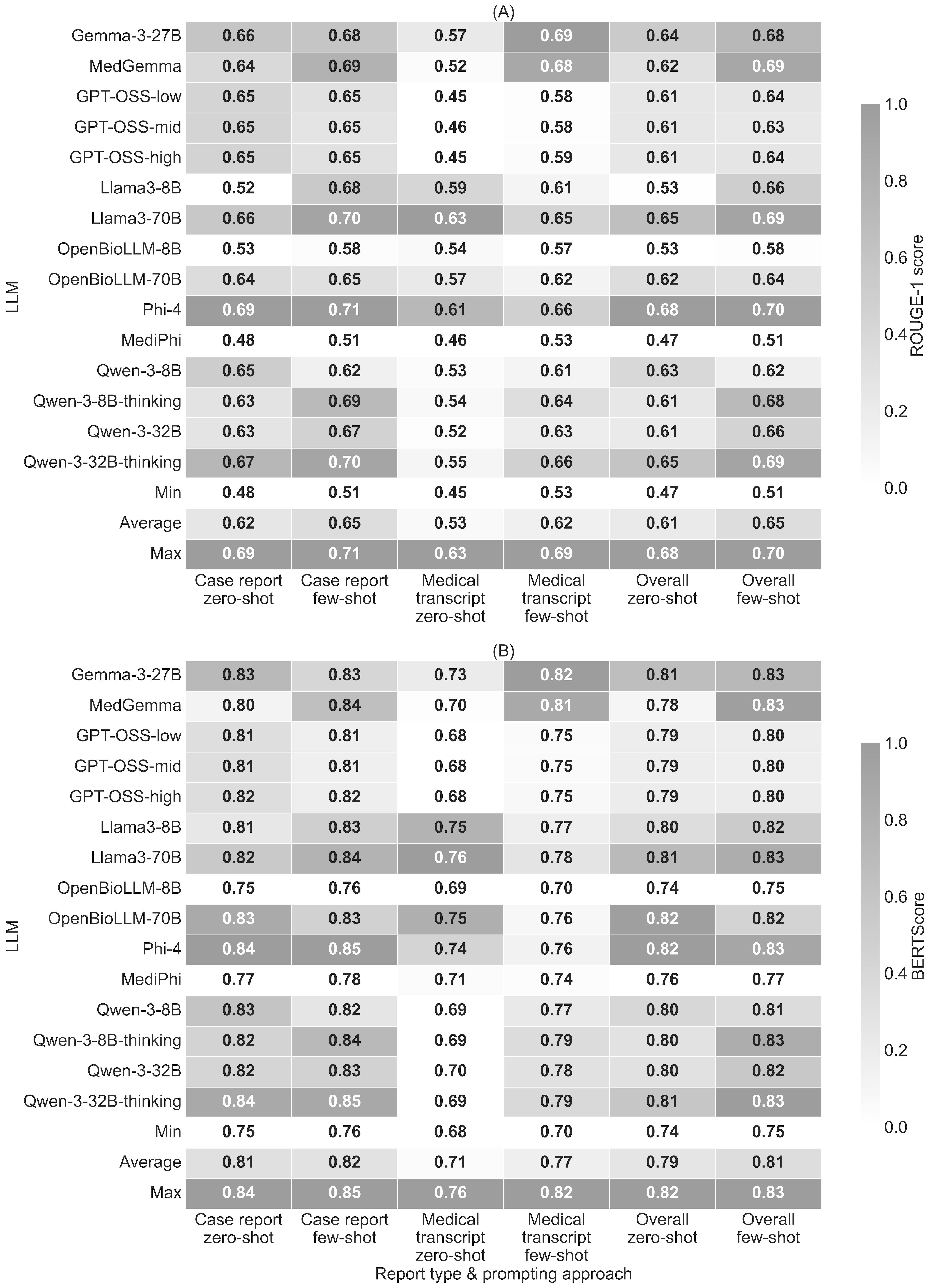

Supplement: ocag070_Supplementary_Data [file ocag070_supplementary_data.zip › Figure A5.png]

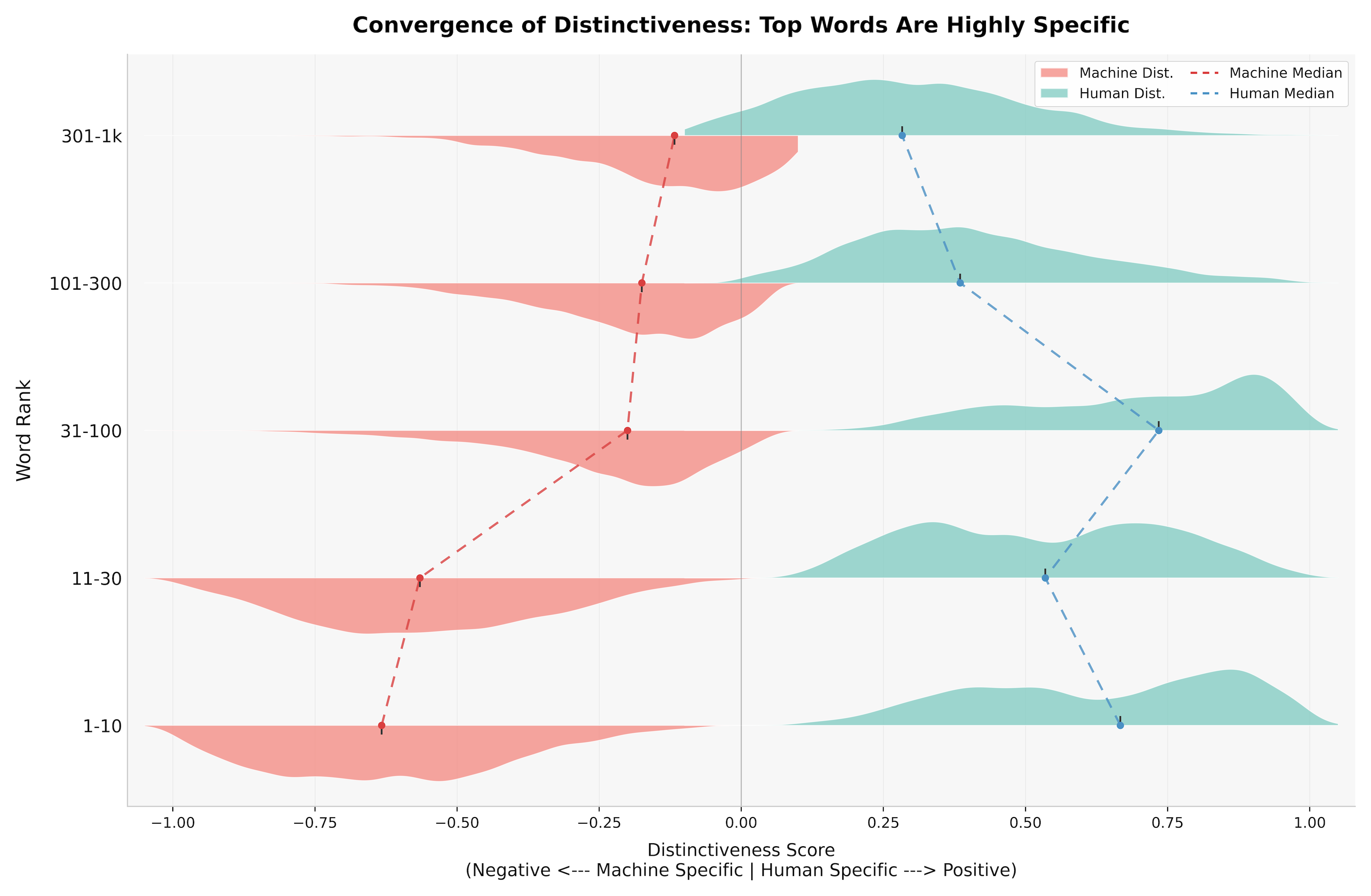

Supplement: ocag070_Supplementary_Data [file ocag070_supplementary_data.zip › Figure A6.png]
